# Lycorine reduces mortality of human enterovirus 71-infected mice by inhibiting virus replication

**DOI:** 10.1186/1743-422X-8-483

**Published:** 2011-10-27

**Authors:** Jiangning Liu, Yajun Yang, Yanfeng Xu, Chunmei Ma, Chuan Qin, Lianfeng Zhang

**Affiliations:** 1 Key Laboratory of Human Diseases Comparative Medicine, Ministry of Health; Institute of Laboratory Animal Science, CAMS & Comparative Medicine Centre, PUMC, Chao Yang Strict, Pan Jia Yuan Nan Li No.5, Beijing 100021, China; 2 Key Laboratory of Human Diseases Animal Models, State administration of Traditional Chinese Medicine; Institute of Laboratory Animal Science, CAMS & Comparative Medicine Centre, PUMC, Chao Yang Strict, Pan Jia Yuan Nan Li No.5, Beijing 100021, China

**Keywords:** Human enterovirus 71, Lycorine, Mouse model, Drug

## Abstract

Human enterovirus 71 (EV71) infection causes hand, foot and mouth disease in children under 6 years old and this infection occasionally induces severe neurological complications. No vaccines or drugs are clinical available to control EV71 epidemics. In present study, we show that treatment with lycorine reduced the viral cytopathic effect (CPE) on rhabdomyosarcoma (RD) cells by inhibiting virus replication. Analysis of this inhibitory effect of lycorine on viral proteins synthesis suggests that lycorine blocks the elongation of the viral polyprotein during translation. Lycorine treatment of mice challenged with a lethal dose of EV71 resulted in reduction of mortality, clinical scores and pathological changes in the muscles of mice, which were achieved through inhibition of viral replication. When mice were infected with a moderate dose of EV71, lycorine treatment was able to protect them from paralysis. Lycorine may be a potential drug candidate for the clinical treatment of EV71-infected patients.

## Introduction

Human enterovirus 71 (EV71) is a positive-stranded RNA virus belonging to the enterovirus genus of the Picornaviridae family [1]. EV71 infection predominantly leads to hand, foot and mouth disease (HFMD) in children under 6 years old [2,3]. Most of the EV71 infections resolve spontaneously; however, EV71 infection occasionally causes neurological complications that can lead to cardiopulmonary failure and death [4-7]. Outbreaks of EV71 have erupted around the world in the past decades and mainly appearing in the countries and regions of Asia in recent years [8-10]. Hundreds of cases involving lethal complications have been reported in each outbreak [2,11,12], and the epidemic is still prevalent in Asia [13].

Currently, there are no vaccines or antiviral drugs available to use against EV71 infection in the clinic, and the prevention of EV71 epidemics depends upon public surveillance alone. In recent years, there have been many efforts to develop drugs to combat EV71 infection and dozens of drugs have been reported to show anti-EV71 activity in vitro, some of which have been evaluated in animal models including bovine lactoferrin [14], ribavirin [15], siRNA [16] and type I interferon [17]. Although these drugs showed activity against EV71 infection both in cell lines and in animal models, the clinical application is not yet available.

Lycorine is one of the most abundant alkaloids of Amaryllidaceae [18], and has a wide range of biological effects including apoptotic effects on tumor cells [19], antimalarial effects [20], anti-inflammatory effects [21] and induction of nausea and emesis [22]. Importantly, lycorine also has been shown to have antiviral effects on human immunodeficiency virus (HIV-1), severe acute respiratory syndrome-associated coronavirus (SARS-CoV), poliovirus, coxsackie virus, measles virus, and herpes simplex virus type 1 [23-25].

In the present study, the activity of lycorine against EV71 replication was investigated in vitro and in mice models infected with EV71 strain.

## Materials and methods

### Cells and viruses

Human rhabdomyosarcoma cells (RD) were maintained in Dulbecco's modified Eagle's medium (DMEM) containing 10% fetal bovine serum (FBS) as previously described [26]. A clinically isolated EV71 strain FY0805 (GenBank accession no. HQ882182) and the mouse-adapted EV71 strain MP10 (GenBank accession no. HQ712020) derived from FY0805 were cultured in RD cells. The virus titres were determined using a plaque assay as described [27], and working stocks of virus containing10^9 ^TCID_50 _/ml were prepared for experiments.

### Antiviral assay in RD cells

For the antiviral assay, RD cells (2 × 10^4 ^cells/well) were plated in 96-well plates with DMEM medium lacking antibiotics and grown overnight to 90% confluence at 37°C. The RD cells were then infected with 100 TCID_50 _of FY0805 and cultured continually in DMEM medium containing 2% FBS. The infected cells were treated with lycorine (purity ≥ 98% in an HPLC assay, National institutes for food and drug control) in a set of concentrations in saline as 0.1, 0.5, 1, 2, 5, 10 and 20 μg/ml. The infected RD cells were observed for CPE or harvested at eight-hour intervals post infection to determine the number of viral RNA copies by quantitative RT-PCR (qRT-PCR). The half maximal inhibitory concentration (IC_50_) was defined as the concentration of lycorine that caused a 50% CPE reduction compared to that of the saline-treated control [14].

### Cytotoxicity Assay

The concentration of lycorine that was required for 50% cell cytotoxicity (CC_50_) was determined in RD cells as described previously [28]. Briefly, RD cells were plated in 96-well plates and grown overnight to 90% confluence at 37°C. The cells were then treated with lycorine for 72 hours at 37°C in a set of concentrations between 1 and 200 μg/ml. CC_50 _was defined as the concentration of lycorine that caused 50% CPE of RD cells.

### Viral proteins expression assay by western blotting

To assess the expression of viral proteins following lycorine treatment, RD cells (2 × 10^4 ^cells/well) were plated in 96-well plates and grown overnight to 90% confluence at 37°C. The cells were then infected with 10^5 ^TCID_50 _of FY0805 and cultured continually for 6 hours. The infected cells were treated with 1 μg/ml lycorine. The infected RD cells were harvested after washing three times with PBS (pH 7.2, Gibco) at 0.5, 1.0 and 1.5 hours of treatment. The harvested cells were resuspended in 20 μl of PBS and lysed with three freeze-thaw cycles and the cell debris was then removed by centrifugation at 3,000 g for 20 minutes. The supernatants were subjected to viral RNA and protein analysis, and the infected RD cells treated with saline were used as control.

For western blotting assay, equal amounts of aliquots (20 μl of supernatants) were separated by 12% SDS-PAGE and transferred onto a nitrocellulose membrane (Immobilon NC, Millipore France). The polyclonal mouse antibodies against the viral peptides were prepared in our lab (anti-P_70-159 _for VP2, anti-P_324-443 _for VP3, anti-P_566-665 _for VP1, anti-P_1329-1440 _for 2C, anti-P_1649-1731 _for 3C, and anti-P_1843-1951 _for 3D, 1:1000 dilution) [29]. Primary antibodies were visualised with HRP-conjugated goat anti-mouse secondary antibodies (Sigma) using a chemiluminescent detection system (Santa Cruz). The bands were quantified by densitometry using Quantity One software. The intensity of bands for six viral proteins in the saline-treated group were used as standards and defined as 100. The density of the bands of viral proteins in the lycorine-treated group was compared to their respective standards.

### Determination of the viral load

qRT-PCR was used to detect the viral RNA copy number. Briefly, total RNA was isolated from cultured cells or tissues from mice using the TRIzol reagent. The total RNA was then reverse transcribed using random hexamers with a reverse-transcription kit (Promega). The cDNA was subjected to quantitative PCR (QuantiTect SYBR Green RT-PCR kit, QIAGEN) with a Roche Light Cycler3.5 system for 40 cycles. The primers were EV71-S1 (5'-AGATAGGGTGGCAGATGTAATTGAAAG-3') and EV71-A1 (5'- TAGCATTTGATGATGCTCCAATTTCAG-3'). A fragment corresponding to nucleotides 2462-2635 of FY0805 was adjusted to a concentration gradient (1 × 10^1 ^copies/μl to 1 × 10^8 ^copies/μl) and was used as standard to calculate the copy number of viral RNA. Results were normalised to GAPDH.

Semi-quantitative RT-PCR was used to confirm the results of qRT-PCR. Briefly, the viral cDNA obtained above was assayed by PCR amplification with the same primers, and the results were normalised to GAPDH.

### Mouse protection assay

Ten- or eleven-day-old ICR mice were provided by the Institute of Laboratory Animal Science, Peking Union Medical College. All of the animal protocols were approved by the institutional animal care and use committee. For lethal EV71 challenge, each ten-day-old mouse was intraperitoneally (i.p.) inoculated with 1 × 10^7 ^TCID_50 _(lethal dose) of MP10. For self-limited model development, each mouse at eleven-day-old was inoculated with 1 × 10^6 ^TCID_50 _of MP10 via the i.p. route. At 12 hours post infection, the infected mice were intraperitoneally injected with different concentrations of lycorine in saline daily twice for 7 days. The placebo group was injected with the same volume of saline as control. The symptoms and survival rates of infected mice were monitored daily for 2 weeks, the clinical scores were graded following previously described standards [15] and the muscle tissues of mice were sampled at 1 dpi, 3 dpi, 5 dpi, 7 dpi and 9 dpi and sent for virology and pathology analysis.

### Pathology

For each experimental group, six mice were subjected to pathologic examination. After euthanasia, the muscle tissues were immediately immersion-fixed in 10% buffered formalin for 48 hours. The tissues were bisected, embedded in paraffin, and stained with hematoxylin and eosin stain (H&E). Ten sections of muscle were observed per animal in a blinded manner.

### Immunohistochemistry

Immunohistochemistry (IHC) was used to detect virions in the infected tissues as described previously [26]. Briefly, the sections were incubated with a monoclonal mouse antibody for VP1 of EV71 (Millipore, 1:200 dilution) for 1 hour at 37°C. The sections were then washed three times with PBS and incubated with HRP-conjugated goat anti-mouse IgG (1:5000 dilution, Sigma) for 1 hour at 37°C. The sections were developed with 3-3'diaminobenzidine (DAB) and observed under a light microscope (Olympus).

### Statistics

All data are expressed as the mean ± SD. The statistical significance of differences in mean values was assessed by Duncan's multiple-range test following a one-way analysis of variance (ANOVA), and survival rates were analysed by Kaplan-Meier analysis. A P value of < 0.05 was considered to be significant.

## Results

### Lycorine inhibits EV71 infection in RD cells

**Figure 1:**
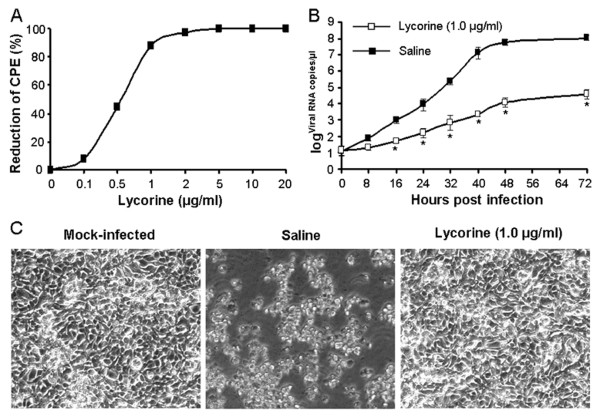
**The inhibition effect of lycorine on RD cells**. A, IC_50 _of lycorine for inhibiting EV71 infection was determined by the reduction of CPE on RD cells. B, the viral copy number was detected by qRT-PCR in the RD cells treated with lycorine at a dose of 1 μg/ml. The data are expressed as mean values of three independent experiments ± SD, *: p < 0.05. C, CPE of EV71-infected RD cells at 3 dpi were observed under light microscope (100×).

**Figure 2:**
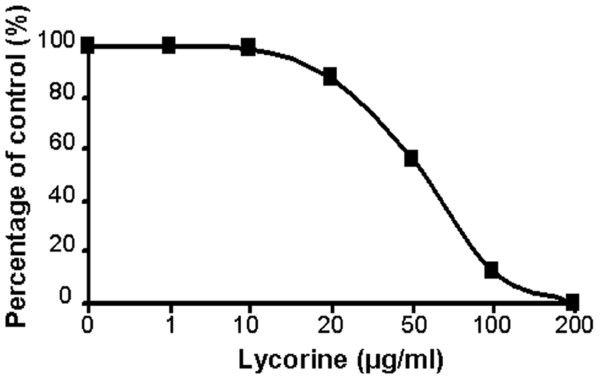
**Determination of the CC_50 _of lycorine on RD cells**. The RD cells were seeded into the 96-well plate and culture for 24 hours. Lycorine with different concentrations were added in the wells and cultured continually for 3 days. The CC_50 _was calculated by the CPE of RD cells. The data are expressed as mean values of three independent experiments.

The anti-EV71 efficacy of lycorine was tested on a human muscular (RD) cell line in a plaque reduction assay. As a positive control, the IC_50 _of ribavirin was determined in present study and was 60 μg/ml [15]. Similarly, lycorine inhibited EV71 infection of RD cells in a dose-dependent manner with an IC_50 _of 0.48 μg/ml (Figure 1A). And the replication of EV71 (Figure 1B) and majorities of the CPE in RD cells (Figure 1C) were significantly inhibited by treatment with 1.0 μg/ml lycorine. The CC_50 _of lycorine (48.5 μg/ml, Figure 2) on RD cells was approximately 100-fold higher than the IC_50 _of it against EV71 infection_._

### Lycorine blocked the elongation of the viral polyprotein during translation

**Figure 3:**
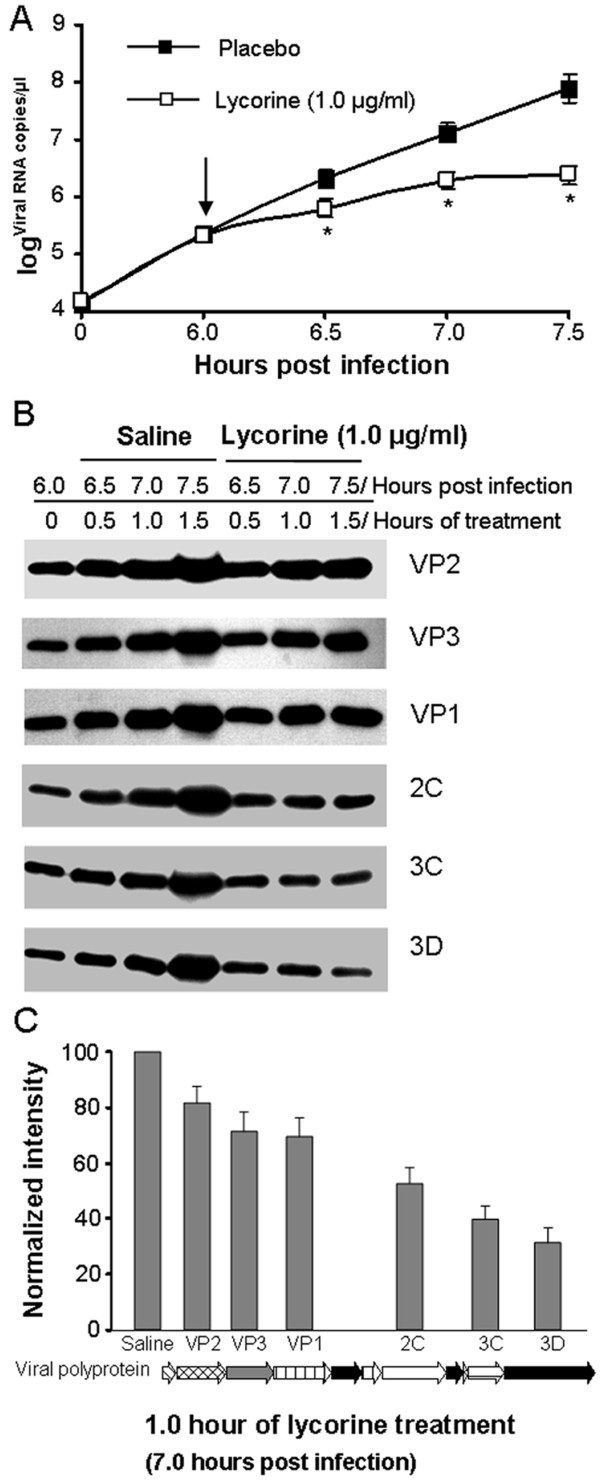
**Lycorine inhibited the elongation of EV71 polyprotein**. The RD cells were cultured in 96-well plate and infected with EV71 virus. The cells were treated with or without lycorine (1 μg/ml) at 6 hours post infection. A, the Viral RNA copies in RD cells were determined by qRT-PCR in a 0.5 hour intervals for 1.5 hours of treatment and the early stage of lycorine inhibition for virus was detected (*: p < 0.05); B, in the early stage of virus inhibition (0.5, 1.0 and 1.5 hours of lycorine treatment), the amounts of viral proteins in infected RD cells was detected by western blotting with the specific primary antibodies against different viral proteins. C, the intensity of bands of viral proteins in control cells were defined as 100, and the intensity of bands of viral proteins in lycorine-treated cells were compared respectively with those of saline-treated cells at 1 hour of treatment. These data are expressed as mean values of three independent experiments ± SD.

To study the inhibitory mechanism of lycorine against EV71 infection, the synthesis of several typical viral proteins were detected by western blotting in 1.5 hours periods following lycorine treatment, when the effects of inhibition was just appeared (Figure 3A and 3B). VP2, VP3, VP1, 2C, 3C and 3D were sequentially translated from N-terminal to C-terminal of the viral polyprotein during viral proteins synthesis. The band densities of these proteins at 1.0 hour of saline-treatment were set as 100. Then the densities of these proteins at 1.0 hour of lycorine-treatment were compared with that of saline-treatment respectively (Figure 3B and 3C). The inhibition of lycorine on synthesis of C terminal proteins was more remarkable than that of N terminal proteins, as the inhibitory rate of VP2 was 12.7%, whereas that of 3D was 66.5%. This result suggested that lycorine affected the elongation of the viral polyprotein during translation.

### Lycorine reduced the mortality of mice upon lethal EV71 challenge

**Figure 4:**
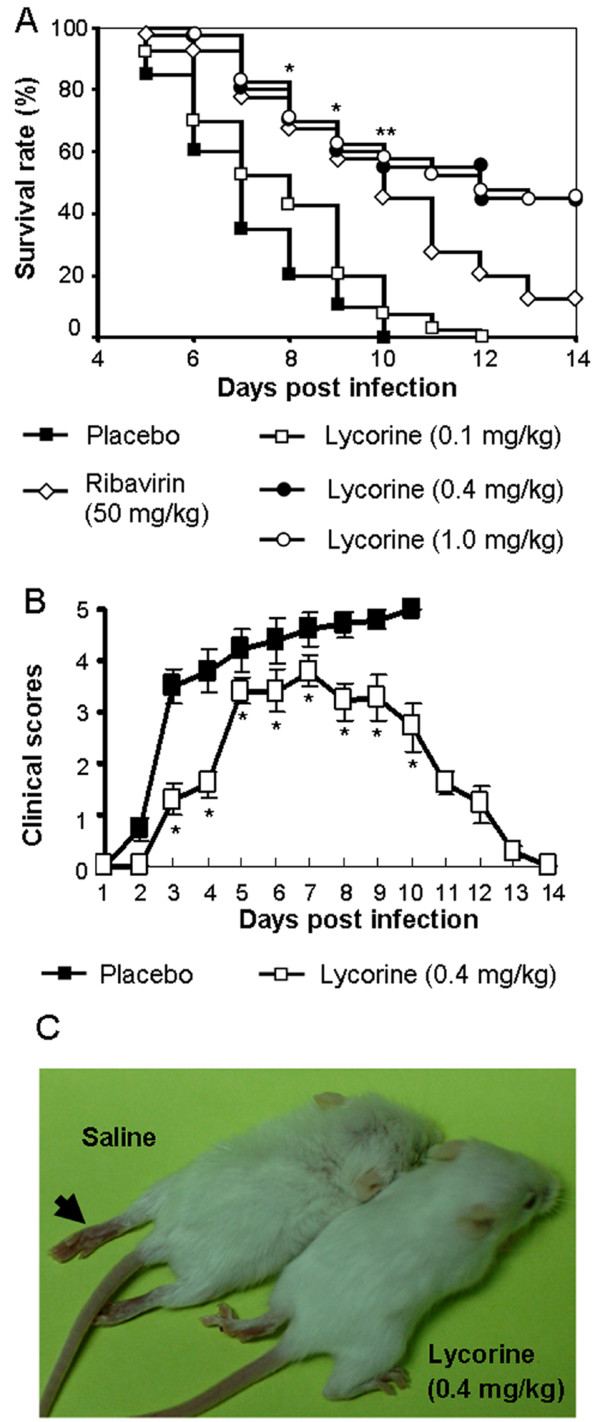
**Lycorine treatment reduced the mortality of EV71-infected mice**. A, survival rates of the EV71 infected mice treated with placebo, ribavirin and lycorine (three different doses) were recorded to14 dpi (n = 40). *: p < 0.05, **: p < 0.001 (Survival rate of the mouse after 0.4 mg/kg lycorine treatment compared to that of the placebo). B, the clinical scores of infected-mice treated with placebo or lycorine (0.4 mg/kg) was systematically evaluated in an independent experiment (n = 40). *, p < 0.05. C, photos were a typical phenotype of ruffled hair and paralysis of hind limbs caused by EV71 infection at 8 dpi (left panel, indicated by arrow) and the phenotype was prevented by the lycorine treatment (right panel).

We utilized the mouse model of lethal EV71 infection to evaluate the effect of lycorine on inhibiting EV71 infection. The placebo-treated mice developed paralysis at 3 dpi and all of them died within 10 dpi, and treatment with ribavirin (50 mg/kg body weight) enhanced the survival rate of infected-mice to 12%. Meanwhile, the lycorine-treatment at doses of 0.1 mg/kg prolonged the survival time of mice; while the treatment at doses of 0.4 or 1.0 mg/kg enhanced the survival rates of mice to 45% respectively (Figure 4A). This result revealed that 0.4 mg/kg was a proper dosage and this dosage was used to analysis the effect of lycorine against EV71 in the further experiments.

**Figure 5:**
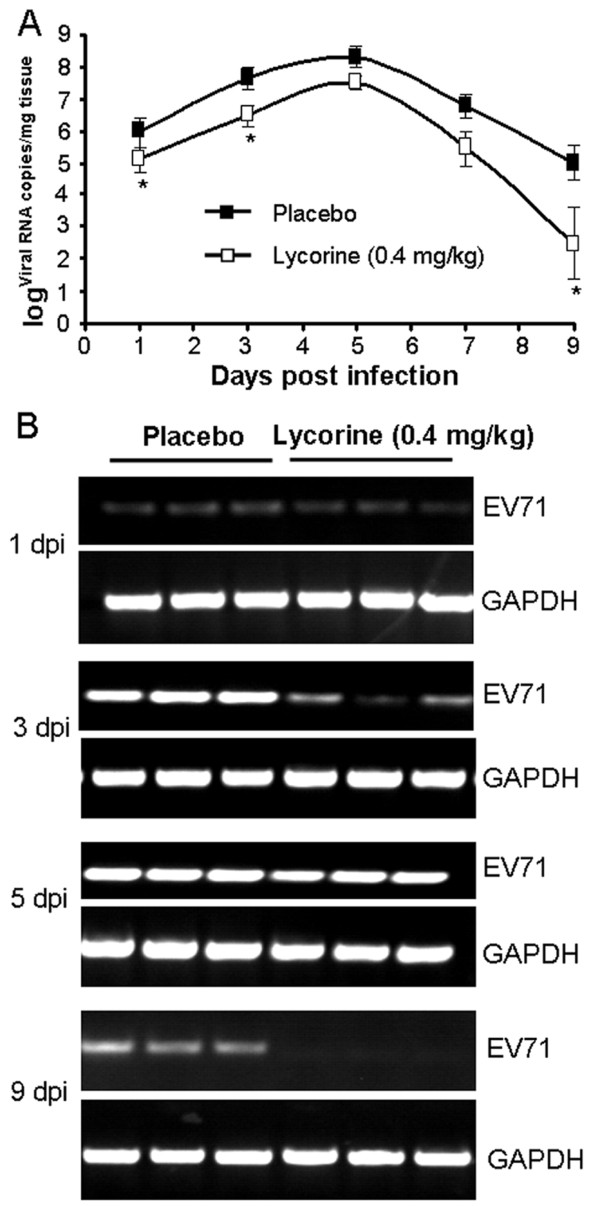
**Lycorine inhibited the replication of EV71 in muscle tissues of mice**. The infected-mice were treated with placebo or lycorine at a dose of 0.4 mg/kg. The muscle tissues were sampled at 1 dpi, 3 dpi, 5 dpi and 9 dpi respectively (n = 8). A, the viral replication curve were determine by qRT-PCR (*: p < 0.05). B, the viral burden was confirmed by semi-quantitative RT-PCR.

**Figure 6:**
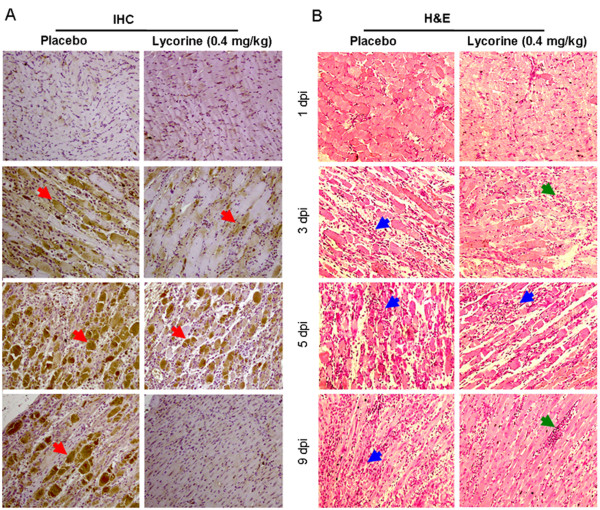
**Lycorine reduced the virus distribution and the pathological damages**. The infected mice were treated with placebo or lycorine at a dose of 0.4 mg/kg. The muscle tissues were sampled at 1 dpi, 3 dpi, 5 dpi and 9 dpi for pathological analysis. A, the virus distribution was detected by immunohistological staining. B, the pathological changes were observed after H&E staining. The red arrows indicated virions in tissues, the green arrows for moderate inflammation and the blue for necrotising myositis. Magnification: 100×.

The clinical scores of infected mice treated with placebo or lycorine (0.4 mg/kg) were systematically evaluated. Treatment with lycorine delayed the paralysis appearance to 1 day later compared with that of the placebo. And the surviving mice (45%) in the lycorine group were completely recovered within 14 dpi (Figure 4B and 4C), while all of the mice in the placebo group were died within 10 dpi. Consistent with the results of the clinical scores and survival rates, virus replication in the muscle of lycorine-treated mice were inhibited by 10-100 folds at different time points compared to that of the saline control as detected by qRT-PCR and semi-quantitative RT-PCR (Figure 5A and 5B). Lycorine treatment also obviously reduced the amount of virions in muscle tissues compared to the saline control by immunohistological staining (Figure 6A). In the saline-treated group, serious muscle necrotic appeared at 3 dpi and lead to paralysis of mice, while the lycorine-treatment (0.4 mg/kg) obviously reduced the muscle damage caused by EV71 infection (Figure 6B, moderate inflammation were observed in the muscle tissues of lycorine treated mice at 3 dpi and 9 dpi, contrast to the necrotising myositis of placebo-treated mice from 3 dpi to 9 dpi).

### Lycorine treatment protected mice from apparent symptoms post non-lethal dose EV71 challenge

**Figure 7:**
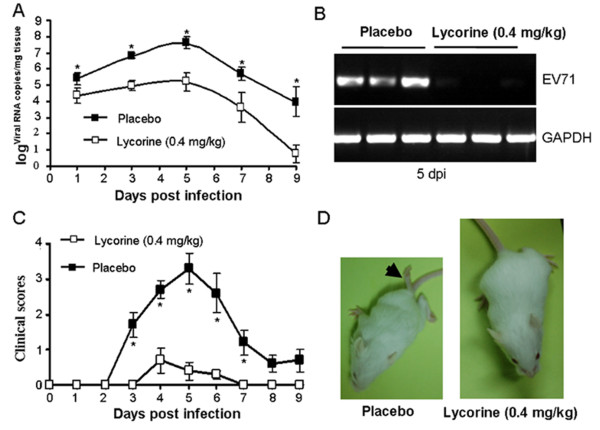
**The effect of lycorine on self-limited mouse model**. The self-limited mouse model was established with a moderate dose of MP10 infection as described in the methods. The infected-mice were treated with placebo or lycorine at a dose of 0.4 mg/kg. The muscle tissues were sampled at 1 dpi, 3 dpi, 5 dpi and 9 dpi respectively (n = 6). A, the viral replication curve were determine by qRT-PCR (*: p < 0.05). B, the viral burden was confirmed by semi-quantitative RT-PCR. C, the clinical scores of the infected mice treated with placebo or lycorine were recorded (n = 20). *: p < 0.05. D, photos were a typical transient phonotype of ruffled hair and hind limb paralysis caused by a moderate dose of EV71 infection at 5 dpi (left, indicated by arrow) and the phenotype was prevented by the lycorine treatment (right panel).

The mouse model with lethal-dose EV71 infection was used to imitate the severe complications of EV71 infected patients. However, as most of the EV71 infection in patients leads to symptoms resolves spontaneously [13], we also explored the effect of lycorine against a moderate dose of MP10 infection in mice, which was supposed to imitate the self limited patients. The virus replication in muscle tissues of lycorine-treated (0.4 mg/kg) mice was significantly inhibited by more than 100-fold compared to the saline control as detected by qRT-PCR and semi-quantitative RT-PCR (Figure 7A and 7B). The saline-treated mice developed transient ruffled hair and paralysis and recovered within 8 days. The lycorine treatment protected the infected-mice from symptoms of paralysis except occasionally skin fur (Figure 7C and 7D). These results indicated that lycorine treatment was able to completely protect the mice from obviously symptoms upon a low-dose EV71 challenge.

## Discussion

Along with its broad biological activities and capacity to inhibit the replication of many different viruses, we confirmed that lycorine can inhibit EV71 infection. Lycorine showed inhibitory activity against EV71 replication in RD cells, and lycorine treatment significantly enhanced the survival rate of EV71-infected mice, which demonstrates that lycorine may be a potential drug candidate for the clinical treatment of EV71 infection-induced diseases.

Previous studies indicated that lycorine can inhibit protein synthesis in eukaryotic cells [30,31] and in cell-free systems in which protein synthesis is catalysed by eukaryotic ribosomes [32]. However, there have debates on which step of protein synthesis is blocked by lycorine. Jimenez et al. has indicated that lycorine halts protein synthesis in eukaryotic cells by blocking the peptide bond formation [31]. Lycorine was shown to prevent the coupling of the N-acetyl leucyl residue from UACCA-acetyl leucine to puromycin, and it was therefore postulated that these compounds inhibited the transpeptidation reaction [32]. In contrast, it has been reported that lycorine affects the termination but not the elongation nor the cleavage of the polyprotein [33]. The single large coding region of the EV71 genome is flanked by 5' and 3' untranslated regions (5' and 3' UTR). The coding region is translated as a single polyprotein, which is then processed by viral proteases to yield mature viral proteins including the capsid proteins (VP1-VP4) and non-structural proteins (2A-2C and 3A-3D) [34]. We investigated the inhibitory mechanism of lycorine on EV71 replication by detecting the step of the viral life cycle that was initially blocked post drug treatment and found that the repression effect of lycorine on the synthesis of viral proteins located at C terminal of the polyprotein was earlier and more substantial than its effect on viral proteins located at N terminal of polyprotein (Figure 3); therefore, we concluded that the drug inhibits the elongation of the viral polyprotein during protein synthesis. The imbalanced synthesis of viral proteins could interrupt the package of the virus. And the inhibition of 3D protein could result in the reduction of replication of virus, as the 3D protein is the RNA polymerase of EV71 [35].

In contrast to its other wide range of biological effects, lycorine is suspected to be the source of Amaryllidaceae poisoning in humans and animals [36]. Empirical data from previous reports suggests that lycorine may be responsible for symptoms such as nausea and emesis [22]. High concentration of lycorine-induced apoptosis of RD cells was observed in this study (Figure 2), which indicated that lycorine is noxious to eukaryotic cells. However, lycorine could inhibit the replication of EV71 at very low concentration and the IC_50 _for inhibiting EV71 infection was approximately 100-fold lower than the CC_50 _on RD cell, suggesting that it had potential application in the clinical treatment of EV71 infection. This assumption was verified by evaluating the antiviral effect of lycorine in a mouse model, as the survival rate of 0.4 mg/kg lycorine-treated mice was significantly enhanced compared to the control mice upon lethal EV71 infection (Figure 4A), and the alleviation of acute symptoms and reduction of pathological changes in the muscles of lycorine-treated mice were achieved by inhibiting the replication of virus (Figure 4, 5, 6). The surviving mice in the lycorine treated group recovered within 14 days. Furthermore, lycorine treatment did not cause any obvious side effects in the mice at the tested doses (data not shown).

Following lethal EV71 infection, the lycorine-treated mice had a survival rate that reached 45%, while all of the saline-treated mice were died within 14 dpi (Figure 4). In previous studies, treatment with several drug candidates, including ribavirin, bovine lactoferrin, siRNA and type I interferon, were able to enhance the survival rates of infected mice to 18%-100% [14-17]. However, because the infection doses of virus and strains were different in these experiments, it is difficult to compare the activity of these drugs, but we observed that the efficiency of lycorine against EV71 infection was better than ribavirin, as shown in IC_50 _determination (Figure 1A) and evaluation by a mouse model (Figure 4A). Furthermore, as most of the EV71 infections were self-limited and did not cause severe complications in patients, self-resolved EV71 infection model, in which the infected mice develop paralysis at 5 dpi and the symptoms were basically self-limited within 8 dpi. As we expected, lycorine treatment was able to protect the infected-mice from paralysis symptoms by inhibiting EV71 replication (Figure 7). Meanwhile, we found that lycorine could also inhibit the infectivity of three other EV71 strains (these strains were isolated from clinical specimens from different regions of mainland China and belong to the C4 genotype) on RD cells (data not shown); which demonstrates that lycorine has an effective antiviral spectrum against EV71 strains prevalent in mainland China.

## Conclusion

In summary, we have shown that lycorine is a promising drug candidate for treating EV71 infections that has potential application in the clinical therapy of EV71-infected patients and that may contribute to the control of EV71 epidemics in Asia.

## Competing interests

The authors declare that they have no competing interests.

## Authors' contributions

JL and YY carried out all experiments except for the pathology analysis and draft the manuscript. YX and CM carried out pathology analysis and immunohistochemical staining. CQ participated in the designing of experiment and edited the manuscript. LZ provided overall supervision, financial support and prepared the final version of the manuscript. All authors read and approved the final manuscript.
